# Inflammation of the Dental Pulp

**DOI:** 10.1155/2015/980196

**Published:** 2015-12-30

**Authors:** Sang Hyuk Park, Ling Ye, Robert M. Love, Jean-Christophe Farges, Hiromichi Yumoto

**Affiliations:** ^1^Department of Conservative Dentistry, College of Dentistry, Kyung Hee University, Seoul 02447, Republic of Korea; ^2^The State Key Laboratory of Oral Diseases, West China School of Stomatology, Sichuan University, Sichuan 610042, China; ^3^Department of Restorative Dentistry, University of Otago, School of Dentistry, Dunedin 9016, New Zealand; ^4^Department of Oral Biological Sciences, University Lyon 1, Faculty of Odontology and Laboratory of Tissue Biology and Therapeutic Engineering, UMR5305 CNRS/University Lyon 1, 69372 Lyon, France; ^5^Department of General Dentistry, Tokushima University Hospital, Tokushima 770-8503, Japan

The dental pulp is sensitive to external factors such as microbial infection from dental caries and/or mechanical/chemical irritations during dental procedures. Dental tissue behaves differently to the other connective tissues. It is unique in a way that its soft tissues (pulp and pulp-dentin complex) are enclosed within mineralized hard tissues (enamel, dentin, and cement), and its pulp is supplied by a rich neurovascular network that regulates various inflammatory mediators [1, 2]. Inflammatory signals may progress to rapid degeneration and necrosis, and such events could inflict very serious damage to tissues in the body [3, 4].

Factors that induce inflammation in the dental pulp and the root apex are as follows [1, 2, 5, 6]: the ingression of microorganisms through dental caries, crack or dentinal tubules of the teeth; chemical irritation from etching and/or bonding materials for adhesion of dental materials; mechanical irritation during preparation in restorative procedures; trauma from occlusion (TFO) or orthodontic movement of the teeth. Such factors may initiate the inflammatory cascades, which, in turn, further progress to pain and root resorption via neurogenic inflammation and hard tissue remodeling [7–9]. Therefore, a thorough understanding of the pulpal inflammatory process is essential in the development of proper dental procedures and immunotherapeutic agents.

This special issue is published with the intent of disseminating current knowledge and findings on inflammation in the dental pulp. This issue highlights the molecular mechanisms of inflammatory cascades, immunomodulatory effects of various substances, techniques used to study inflammation and regeneration of the dental pulp, and ultimately why an understanding of inflammatory process is important in the field of endodontics.

In this issue, J.-H. Jang et al. review pathogen recognition receptors (PRRs) for innate immunity in dental pulp. PRRs recognition of pathogen-associated molecular patterns (PAMPs) on pathogenic structure is important stage of initiating specific adaptive immunity. The paper reviews the various types of PRR families that include the Toll-like receptors (TLRs), the C-type lectin receptors (CLRs), the nucleotide-binding oligomerization domain-like receptors (NLRs), the retinoic acid-inducible gene-I-like receptors (RLRs), and the AIM2-like receptor (ALR). The authors suggest that immunomodulation via PRRs is crucial in the understanding of pathophysiology of pulp inflammation and also in the development of novel therapeutic targets in the pulp injury and/or infection.

J.-C. Farges et al. assess responses of the pulp tissues to bacterial infection at cellular and molecular levels. The pulp response is illustrated by mechanisms used by odontoblasts and specialised immune cells to resist pathogenic bacteria. The cytokine IL-8 which are upregulated in response to bacterial component is particularly important, because it is involved in the recruitment and activation of neutrophils at the site of inflammation. This paper reports that the balance between the inflammatory response and the repair process is of paramount importance, as they dictate the rate of pulpal healing.

Y.-J. Ko et al. study the anti-inflammatory effect of human telomerase-derived peptide (GV1001) stimulated by lipopolysaccharide (LPS) from* P. gingivalis*. Confocal microscopy was used to analyse the intracellular distribution of GV1001, and real-time RT-PCR was performed to quantify the levels of TNF-*α* and IL-6 cytokines. The study reports that GV1001 was capable of entering the cell and caused downregulation of LPS-induced inflammatory cascades demonstrating its potential as a therapeutic agent in vital pulp therapy, regenerative endodontic fields, and tissue engineering.

S. Kim et al. review in vivo experiments with dental pulp stem cells to observe pulp-dentin complex regeneration. Most studies in this area have been conducted with mouse or dog models making their application in clinical environment unreliable. The authors suggest that where clinical trial of a study is impractical, orthotopic transplantation of dental pulp tissue cells (DPSCs) should be performed, as it would better predict impacts in human teeth. This paper claims that more emphasis in orthotopic transplantation in animal model is required to further expand our knowledge in pulp-dentin complex regeneration.

M. Goldberg et al. discuss the importance of inflammation in the pulp healing and regeneration. Despite its association with undesirable symptoms in pulpitis, inflammation should be understood as prerequisite of the pulp healing. The view is supported by inflammation-mediated proliferation of pulp cells and initiation of mineralisation. Inflammation may also be involved in the production of a reparative dentinal bridge that covers the pulp exposure.

D. Chavarria-Bolaños et al. conduct in vivo experiment to study sensory neuropeptides and endogenous opioids expression in human dental pulp with asymptomatic inflammation. Analysis of substance P (SP), calcitonin gene-related peptide (CGRP), *β*-endorphins (*β*-End), and methionine-enkephalin (Met-Enk) in patients' teeth with asymptomatic inflammation induced by orthodontic intrusion showed the significant increase in the level of SP and CGRP, both of which are frequently associated with pain of pulpal origin.

There is an ever growing interest in keeping teeth in a physiologically functional state in the event of injury and/or infection to the pulp, because no prosthesis can give as much stability and comfort as the normal teeth provide. Inflammatory process that takes place within a tooth is as unique as its structure compared to the other hard tissues in the body. Therefore, an understanding of inflammation of the pulp is a key to control pathophysiological status of the pulp/periapical tissues. This special issue includes reviews and research papers, which help to understand the process of inflammation in the context of dentistry and thereby expand our knowledge to potential therapeutic targets in pulp therapy.

*Sang Hyuk Park* *Sang Hyuk Park* *Ling Ye* *Ling Ye* *Robert M. Love* *Robert M. Love* *Jean-Christophe Farges* *Jean-Christophe Farges* *Hiromichi Yumoto* *Hiromichi Yumoto*
